# B-type natriuretic peptide levels and volume status in twice-weekly hemodialysis patients

**DOI:** 10.1080/0886022X.2021.1971091

**Published:** 2021-08-31

**Authors:** Nina Fang, Miaolin Che, Ling Shi, Zanzhe Yu, Zhaohui Ni, Wei Fang, Huihua Pang, Leyi Gu, Xinghui Lin

**Affiliations:** Department of Nephrology, Renji Hospital, School of Medicine, Shanghai Jiaotong University, Shanghai, China

**Keywords:** B-type natriuretic peptide, volume status, hemodialysis, dialysis frequency, dialysis adequacy

## Abstract

**Background:**

Twice-weekly hemodialysis (HD) could be regarded as an important part of incremental hemodialysis, volume status of this treatment model remains to be elucidated.

**Methods:**

Patients undergoing regular twice-weekly or thrice-weekly hemodialysis in our unit on June 2015 were enrolled into the cohort study with an average of 2.02 years follow-up. Volume status of the subjects was evaluated by clinical characteristics, plasma B-type natriuretic peptide (BNP) levels and bioimpedance assessments with body composition monitor (BCM). Cox proportional hazards models and Kaplan–Meier analysis were used to compare patient survival between the two groups.

**Results:**

Compared with patients on thrice-weekly HD, twice-weekly HD patients had significantly higher log-transformed BNP levels (2.54 ± 0.41 vs. 2.33 ± 0.49 pg/ml, *p* = 0.010). Overhydration (OH) and the ratio of overhydration to extracellular water (OH/ECW) in twice-weekly HD group were significantly higher than that of thrice-weekly HD (OH, 2.54 ± 1.42 vs. 1.88 ± 1.46, *p* = 0.033; OH/ECW, 0.17 ± 0.07 vs. 0.12 ± 0.08, *p* = 0.015). However, subgroup analysis of patients within 6 years HD vintage indicated that the two groups had similar hydration status. Multivariate Cox regression analysis showed that log-transformed BNP levels, serum albumin and diabetes status were predictors of mortality in hemodialysis patients. Kaplan–Meier survival analysis indicated that patients with BNP levels higher than 500 pg/ml had significantly worse survival compared with those with lower BNP levels (*p* = 0.014).

**Conclusions:**

Twice-weekly hemodialysis patients had worse volume status than that of thrice-weekly HD patients especially for those with long-term dialysis vintage, BNP level was a powerful predictor of mortality in HD patients.

## Introduction

In the past few years, an incremental hemodialysis (HD) strategy has been advised through initiation of twice-weekly hemodialysis in order to preserve residual kidney function (RKF) better and to maximize the use of medical resource [1–3]. However, adequacy of twice-weekly HD still remains to be elucidated. The 2006 K-DOQI guidelines recommended that the target of spKt/V at least 2.0 for twice-weekly HD patients with residual native kidney urea clearance (Kr) more than 2 mL/min/1.73 m^2^, and it is not appropriate for those with Kr less than 2 mL/min/1.73 m^2^ selecting this treatment model [4]. Nevertheless, due to financial constraints and resources limitations, it is not uncommon for patients without residual kidney function (RKF) undergoing twice-weekly HD [5–7], and some patients have been reported having treatment with twice-weekly HD for more than 20 years HD vintage [7].

On the other hand, beyond solute removal, adequacy of fluid removal is important for end-stage kidney disease (ESKD) patients. Previous studies on peritoneal dialysis (PD) suggested that it was residual kidney function and volume status rather than peritoneal clearance predicting the outcome of PD patients [8,9]. Hitherto, scarce data are available for the hydration status of twice-weekly HD patients. Here we performed a retrospective cohort study with an average of 2.02 years follow-up, to investigate the volume status of twice-weekly HD patients and compared it with that of thrice-weekly HD patients.

## Materials and methods

### Patient selection

There were 161 patients undergoing routine hemodialysis between 1 June 2015 and 30 June 2015 in Shanghai Renji hospital hemodialysis center (western part). 7 of them were excluded from the study due to their irregular dialysis frequency, 10 of them were also excluded from the study due to incomplete laboratory data, The rest of 144 patients with regular twice-weekly HD or thrice-weekly HD were eligible to be enrolled into the cohort study.

All the patients were dialyzed with Fresenius series dialyzers, most of them used high flux FX80 or FX1000. The hemodialysis modality based on a real-world practice. Most of the patients selected twice-weekly HD due to financial or physical constraints. Follow-up was ended until 1 September 2017. Death was the major end point.

### Data collection

Baseline data on June 2015 were recorded including age, sex, etiology of kidney disease, dialysis frequency, hemodialysis vintage, body surface area (BSA), body mass index (BMI), presence of diabetes mellitus (DM), blood pressure (BP), HD session time, B-type natriuretic peptide (BNP) concentration and other routine biochemical indices, as well as spKt/V and ultrafiltration volume (values taken from Daugirdas formula for calculation of spKt/V). Urine status was also recorded according to patients’ self-description.

Serum samples were collected pre-hemodialysis in the long interval HD session and readily sent for examination, Biochemical tests including hemoglobin, albumin, blood urea nitrogen, serum creatinine, glucose, calcium and phosphorus levels and intact parathyroid hormone (iPTH) were examined using automatic standard equipment. Adequacy of dialysis was estimated by measurement of spKt/V using Daugirdas formula. Weekly Kt/V was counted as spKt/V multiplied by frequency/per week. BNP was measured by an immunoassay system (Access 2, BECKMAN COULTER). Fresenius TDMS software (Therapy data management system, V2.82.4593.33387) was used to assist with data management.

### Bioimpedance spectroscopy with body composition monitor

Patients who had implanted cardiac device or any kind of metal implants were excluded for the bioimpedance analysis, a total number of 97 patients without contraindication were investigated by bioimpedance measurement using body composition monitor (BCM, Fresenius Medical Care GmbH, Bad Homburg, Germany). In view of most patients more than 6 years HD vintage totally losing residual urine output, the BCM study divided the patients into two groups as within 6 years vintage and more than 6 years vintage.

BCM was performed by one trained nurse according to the manufacture’s instruction, the patient was in a supine position pre-hemodialysis in the long interval session. Based on a fluid assessment model, the total body water (TBW), the extracellular water (ECW), the intracellular water (ICW) and the overhydration state(OH) were calculated [10,11]. Percentage overhydration was previously defined as an OH/ECW ratio >0.15 [12].

### Ethical considerations

The study was conducted according to the standards of the Declaration of Helsinki and approved by the Ethics Committee of Renji hospital (No. Renji (2018) 079). All patients provided written informed consent for use of anonymized medical data by investigators.

### Statistical analysis

Statistical analysis was performed using SPSS 19.0 for Windows (SPSS Inc., Chicago, IL). Results were expressed as mean ± SD or median. Comparison of means or percentages were performed using paired and unpaired ANOVA or a Chi–Square test. Correlations were analyzed using Pearson’s correlation coefficient.

The Cox proportional hazards model was used for statistical analysis of survival. Baseline parameters with a *P* value <0.05 in univariate Cox regression were then incorporated into a final multivariate Cox regression analysis. Backward stepwise elimination using the Wald test was applied to remove insignificant variables, a *P* value <0.05 was counted as statistical significance. Kaplan–Meier analysis was used to compare survival between patients with a BNP level greater than 500 pg/ml and those with a lower BNP level. We also performed survival analysis between patients on twice-weekly HD and on thrice-weekly HD.

## Results

### Baseline characteristics

**Figure 1.**
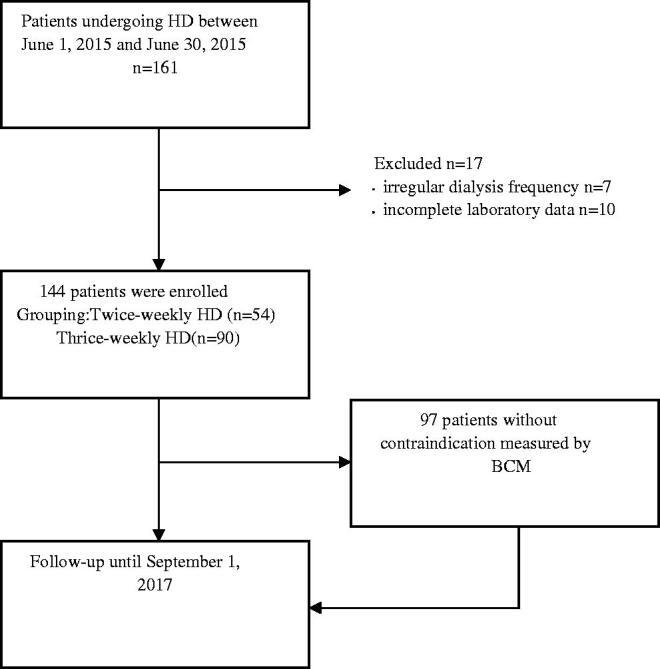
Study design.

144 patients were enrolled in the study (Figure 1), baseline clinical characteristics of the subjects are summarized in Table 1. Briefly, Compared with patients on thrice-weekly HD, the twice-weekly HD patients had significantly longer HD session time but shorter HD vintage (*p <* 0.01). single-pool Kt/V(spKt/V), urea reduction ratio (URR), blood urea nitrogen (BUN) and serum creatinine (Scr) levels in twice-weekly HD patients were significantly higher than that of thrice-weekly HD patients (*p <* 0.01). Systolic blood pressure (SBP) and log-transformed BNP levels in twice-weekly HD were significantly higher as compared with thrice-weekly HD patients (*p <* 0.05).

**Table 1.** Baseline data of 144 HD-patients grouped according to dialysis frequency.

	All patients	Twice-weekly HD	Thrice-weekly HD	*P* value
No. of patients	144	54	90	
Gender *M:F*	75:69	26:28	49:41	
Anuric status, *n* (%)	133 (92.4)	44 (81.5)	89 (98.9)	
Age *year*s	60.2 ± 13.3	61.9 ± 14.6	59.2 ± 12.4	0.243
HD vintage *years*	9.9 ± 6.2	7.7 ± 5.7	11.3 ± 6.1	0.001
Diabetic status, *n* (%)	37（25.7）	13（24.1）	24（26.7）	
Body surface area *m^2^*	1.64 ± 0.18	1.61 ± 0.16	1.66 ± 0.19	0.079
Body mass index *kg/m^2^*	22.0 ± 3.2	21.4 ± 2.8	22.4 ± 3.4	0.077
Systolic BP *mmHg*	143.9 ± 22.7	150.1 ± 18.1	140.2 ± 24.4	0.011
Diastolic BP *mmHg*	75.2 ± 14.1	76.7 ± 11.6	74.4 ± 15.4	0.339
Blood urea nitrogen *mmol/L*	27.8 ± 7.5	32.2 ± 7.9	25.2 ± 5.9	0.000
Serum creatinine *umol/L*	1043.3 ± 275.5	1131.3 ± 279.2	990.6 ± 260.8	0.003
SpKt/V	1.94 ± 0.39	2.11 ± 0.37	1.84 ± 0.36	0.000
URR %	78.3 ± 7.8	81.0 ± 5.2	76.7 ± 8.6	0.001
Weekly SpKt/V	5.03 ± 1.15	4.23 ± 0.74	5.52 ± 1.08	0.000
HD session time *hours*	4.19 ± 0.45	4.44 ± 0.47	4.05 ± 0.38	0.000
UF rate *mL/kg/hr*	13.08 ± 4.00	13.01 ± 4.46	13.11 ± 3.72	0.887
Dry weight *kg*	57.1 ± 11.2	54.7 ± 9.9	58.5 ± 11.7	0.051
Hemoglobin *g/L*	109.1 ± 16.9	107.2 ± 14.5	110.3 ± 18.2	0.295
Serum albumin *g/l*	39.4 ± 3.3	39.7 ± 4.2	39.2 ± 2.6	0.386
HsCRP *mg/l*	4.6 ± 9.0	4.6 ± 10.0	4.7 ± 8.4	0.929
Calcium *mmol/L*	2.29 ± 0.29	2.25 ± 0.27	2.32 ± 0.30	0.193
Phosphorus *mmol/L*	1.99 ± 0.55	2.00 ± 0.54	1.99 ± 0.56	0.990
iPTH *pg/ml*	459.2 ± 407.0	393.0 ± 434.6	498.9 ± 386.5	0.131
Cholesterol *mmol/L*	3.94 ± 0.99	3.96 ± 1.03	3.93 ± 0.98	0.858
Triglyceride *mmol/L*	1.72 ± 1.68	1.37 ± 0.69	1.93 ± 2.03	0.053
BNP *pg/ml*	264.5(14-4995)	346.5(27-4995)	210(14-4995)	
Log BNP *pg/ml*	2.41 ± 0.47	2.54 ± 0.41	2.33 ± 0.49	0.010

NOTE. Data are expressed as mean ± SD, *P* values result from comparisons of baseline values of the two groups divided by HD frequency.

Abbreviations: HD: hemodialysis; BP: blood pressure; SpKt/V: single-pool Kt/V; URR: urea reduction ratio; UF: ultrafiltration; HsCRP: hypersensitive C reactive protein; iPTH: intact parathyroidism hormone; BNP: B-type natriuretic peptide; Log BNP: log-transformed B-type natriuretic peptide.

### Parameters of hydration status with BCM

Table 2 showed the hydration parameters of 97 patients. In brief, when compared with patients on thrice-weekly HD, twice-weekly HD patients showed significantly higher hydration state (OH, 2.54 ± 1.42 vs. 1.88 ± 1.46, *p* = 0.033; OH/ECW, 0.17 ± 0.07 vs. 0.12 ± 0.08, *p* = 0.015), overhydration is predominant in twice-weekly HD patients than that of thrice-weekly HD patients for those more than 6 years HD vintage (*p* < 0.01). However, subgroup analysis of patients within 6 years HD vintage, it suggested that the two groups had similar fluid status (*p* > 0.05).

**Table 2.** Bioimpedance parameters of 97 HD-patients grouped according to dialysis frequency.

	All patients	Twice-weekly HD	Thrice-weekly HD	*P* value
No. of patients	97	36	61	
OH	2.13 ± 1.47	2.54 ± 1.42	1.88 ± 1.46	0.033
<6 years HD vintage	2.02 ± 1.74	2.18 ± 1.51	1.85 ± 2.00	0.592
	(*n* = 33)	(*n* = 17)	(*n* = 16)	
≥6years HD vintage	2.18 ± 1.32	2.86 ± 1.29	1.90 ± 1.24	0.006
	(*n* = 64)	(*n* = 19)	(*n* = 45)	
ECW	14.69 ± 3.01	14.88 ± 2.98	14.59 ± 3.06	0.652
<6 years HD vintage	14.59 ± 3.49	15.03 ± 3.55	14.13 ± 3.47	0.466
	(*n* = 33)	(*n* = 17)	(*n* = 16)	
≥6years HD vintage	14.75 ± 2.77	14.74 ± 2.45	14.75 ± 2.92	0.985
	(*n* = 64)	(*n* = 19)	(*n* = 45)	
OH/ECW	0.14 ± 0.08	0.17 ± 0.07	0.12 ± 0.08	0.015
<6 years HD vintage	0.13 ± 0.09	0.14 ± 0.07	0.12 ± 0.10	0.470
	(*n* = 33)	(*n* = 17)	(*n* = 16)	
≥6years HD vintage	0.15 ± 0.08	0.19 ± 0.07	0.13 ± 0.08	0.004
	(*n* = 64)	(*n* = 19)	(*n* = 45)	

NOTE. Data are expressed as mean ± SD, *P* values result from comparisons of hydration status indexes of the two groups divided by HD frequency.

Abbreviations: OH: overhydration; ECW: extracellular water.

### Correlation analysis

**Figure 2.**
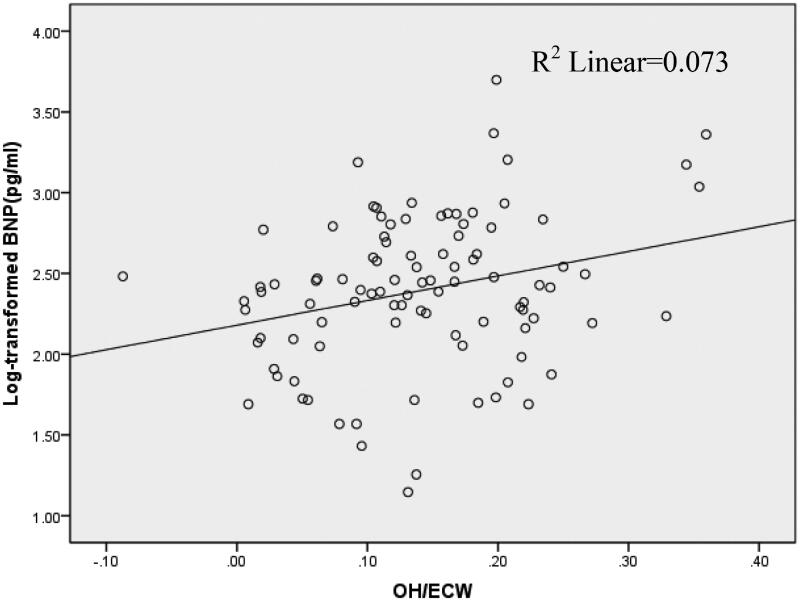
Correlation (*r* = 0.270; *p* < 0.01) between log-transformed brain natriuretic peptide (BNP) and relative overhydration in a cohort of 97 hemodialysis patients.

Correlation analysis showed that log-transformed BNP significantly positively correlated with age, systolic blood pressure, overhydration(OH) and relative overhydration(OH/ECW),but negatively correlated with serum albumin and hemoglobin level (Table 3, Figure 2).

**Table 3.** Correlation analysis between BNP, log-transformed BNP and related parameters.

	Age	SBP	DBP	Hemoglobin	Albumin	HsCRP	OH	OH/ECW
BNP	0.164	0.034	0.080	−0.128	−0.174*	0.171*	0.286**	0.286**
Log-BNP	0.239**	0.187*	0.163	−0.251**	−0.170*	0.061	0.214*	0.270**

NOTE. Analysis performed with Pearson’s product moment correlation.

* Correlation is significant at the 0.05 level (2-tailed).

** Correlation is significant at the 0.01 level (2-tailed).

Abbreviations: BNP: B-type natriuretic peptide; Log BNP: log-transformed B-type natriuretic peptide; SBP: systolic blood pressure; DBP: diastolic blood pressure; HsCRP: hypersensitive C reactive protein; OH: overhydration; ECW: extracellular water.

### Survival analysis

Univariate Cox regression survival analysis is summarized in Table 4. Briefly, age, diabetic status, serum creatinine, hemoglobin, serum albumin, hypersensitive C reactive protein (HsCRP) and log-transformed BNP were each predictors of death (Table 4). In multivariate Cox analysis, only serum albumin, log-transformed BNP and diabetic status were predictors of mortality (Table 5).

**Table 4.** Relative risk (RR) of death in univariate Cox regression for selected variables at baseline in 144 HD-patients during the follow-up.

	RR	95% CI	*P*
Age *1 year*	1.050	1.013-1.088	0.007
Sex *male*	0.847	0.366–1.960	0.697
HD vintage *1 years*	0.993	0.927–1.063	0.834
Diabetic status *present*	4.658	1.990–10.904	0.000
Body mass index *1 kg/m^2^*	1.090	0.963–1.234	0.172
Systolic BP *10 mmHg*	0.934	0.775–1.124	0.469
Diastolic BP *10 mmHg*	0.859	0.661–1.116	0.254
Blood urea nitrogen *1 mmol/L*	0.966	0.911–1.026	0.259
Serum creatinine *100 umol/L*	0.867	0.761–0.987	0.031
Single-pool Kt/V *0.1 unit/per session*	0.948	0.858–1.047	0.289
Weekly SpKt/V *0.1 unit/per week*	0.986	0.952–1.022	0.449
HD session time *1 hours*	0.667	0.303–1.468	0.314
UF rate *100 ml*/*per session*	0.962	0.921–1.005	0.081
Dry weight *1 kg*	1.013	0.977–1.051	0.481
Hemoglobin *1 g/L*	0.975	0.958–0.992	0.004
Serum albumin *1 g/L*	0.777	0.695–0.869	0.000
HsCRP *1 mg/l*	1.029	1.001–1.059	0.044
Log BNP *1 pg/ml*	6.175	2.389–15.958	0.000

Abbreviations: HD: hemodialysis; BP: blood pressure; SpKt/V: single-pool Kt/V; UF: ultrafiltration; HsCRP: hypersensitive C reactive protein; Log BNP: log-transformed B-type natriuretic peptide.

**Table 5.** Multivariate Cox regression analysis of patient survival in 144 HD-patients during the follow-up.

	RR	95% CI	*P*
Serum albumin *1 g/L*	0.812	0.722–0.913	0.001
Log BNP	2.801	1.070–7.332	0.036
Diabetic status *present*	2.859	1.159–7.050	0.023

The original model included age, diabetic status, hemoglobin, serum albumin, hypersensitive C reactive protein(HsCRP) and log-transformed BNP.

**Figure 3.**
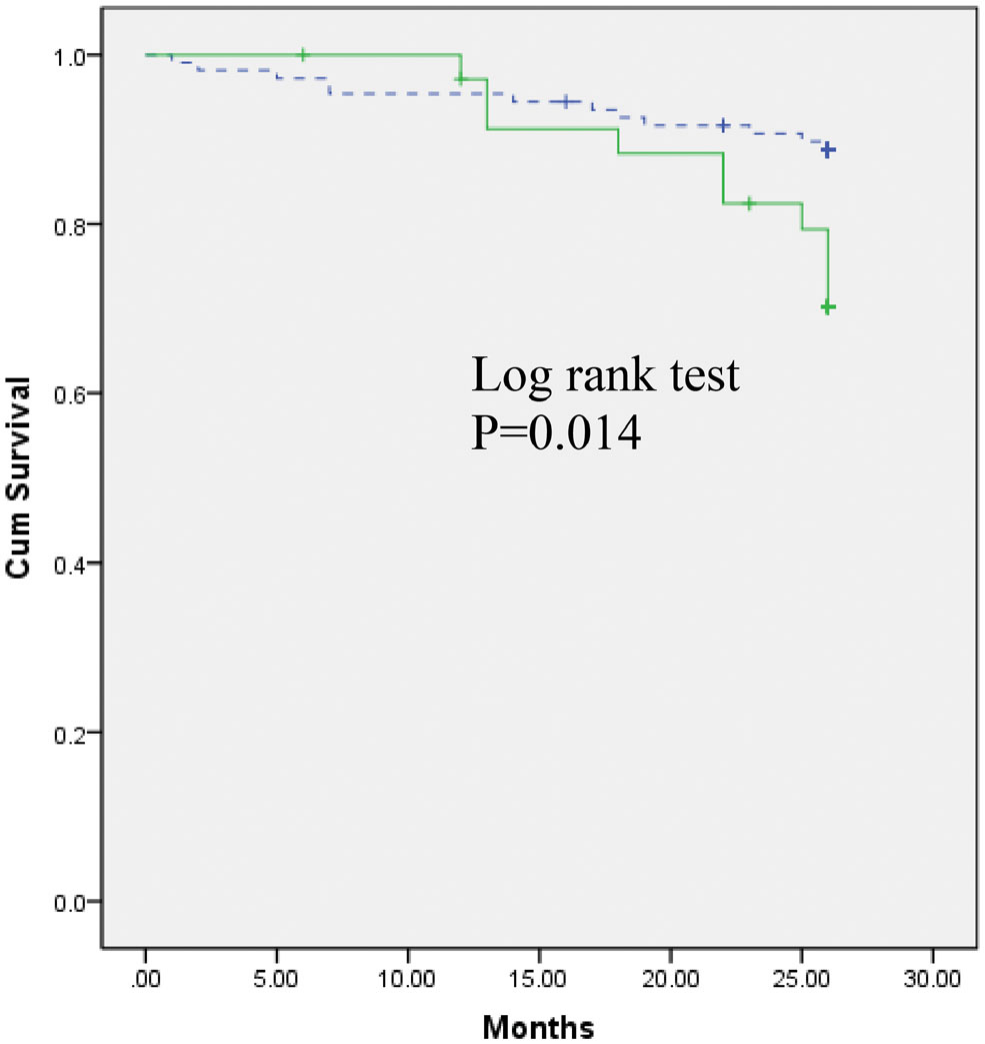
Kaplan–Meier survival curves for all-cause mortality in 144 hemodialysis patients grouped according to BNP ≥500pg/ml (solid) and BNP < 500pg/ml (dashed); *p* = 0.014.

Kaplan–Meier survival analysis indicated that those with a BNP level greater than 500 pg/ml had a significantly worse survival compared with patients with a lower BNP level(*p* = 0.014) (Figure 3). There was no statistical significance of patient survival between twice-weekly HD and thrice-weekly HD groups (*p* = 0.902, Log Rank test).

## Discussion

In the present study, we first reported the volume status of patients with twice-weekly hemodialysis and compared it with those undergoing thrice-weekly hemodialysis. Thus far, adequate assessment of volume status in clinical practice is not an easy task. Traditional clinical tools such as changes in body weight and blood pressure are easy to practice but might be unreliable. In the past few years, several technologies have been developed to assess hydration status in chronic kidney disease (CKD) patients. BNP is a polypeptide secreted by the cardiomyocytes in response to stresses. Thus far BNP and the biologically inactive NT-proBNP have been identified as markers of volume status in CKD patients [13,14], but the cutoff threshold values of BNP for overhydration in ESRD patients remains controversial. Lee and Tapolyai have suggested a value of 500 pg/mL to differentiate between hemodialysis patients with or without volume overload [15,16]. Another important tool to assess fluid status is bioimpedance spectroscopy. This device measures the impedance spectroscopy at 50 frequencies, which enables quantitative assessment of hydration status and body composition. The Body Composition Monitor (BCM) is portable and convenient to practice at bedside. Nowadays BCM has been validated and widely used in clinical practice. Wizemann et al. demonstrated that a relative overhydration (OH/ECW) of more than 15% is related to increased cardiovascular mortality in dialysis patients [12].

Despite that technology advance and great attention has been paid on hydration status, volume overload is prevalent in hemodialysis and peritoneal dialysis patients [17,18]. Devolder reported about 24% of ESRD patients still have clinically relevant volume overload [18]. Previous studies have shown that overhydration was associated with inflammation, malnutrition and left ventricular dysfunction in CKD patients [19–22], and there is a clear relation between volume overload and endothelial dysfunction in patients on renal replacement therapy (RRT) [22]. Hydration status also influenced physical performance, Carlos reported that patients with predialysis fluid overload was associated with slower gait speed and gait speed decline over time [23]. Moreover, cumulative evidence has supported the generally acknowledged idea that volume status is an important predictor of outcome in patients on renal replacement therapy [24,25].

In the present study, we found a correlation between log-transformed BNP level and relevant overhydration. Cox regression analysis showed that log-transformed BNP is an independent predictor of mortality in HD patients. Kaplan–Meier survival analysis indicated that those with BNP level greater than 500 pg/ml had significantly worse survival than patients with lower BNP level. These results corroborated previous findings that fluid status strongly influenced the clinical outcome of dialysis patients [24,25], and it suggested that BNP value of 500 pg/ml could be applied as outcome predictor index for patients on RRT.

Thus far, thrice-weekly hemodialysis has been regarded as standard HD therapy for several decades, but twice-weekly HD is prevalent in the developing countries, and also could be spotted in the United States and in Europe [5,26–28]. The advantages and disadvantages of twice-weekly HD have been discussed in previous studies, accumulated evidence indicated that twice-weekly HD could preserve residual kidney function(RKF) better and might be a cost-effective therapy for patients with certain RKF [1–3,27]. Besides, this treatment model could be counted as an important step for incremental hemodialysis [1–3]. Nevertheless, adequacy of twice-weekly HD remains to be elucidated, and scarce data are available for the hydration status of twice-weekly HD. Hitherto, most studies on fluid status of HD patients were based on thrice-weekly HD [13,18]. In the present study, we first reported the volume status of patients with twice-weekly hemodialysis. We found that twice-weekly HD patients tended to have worse volume status as compared with that of thrice-weekly HD with time. Interestingly, subgroup analysis of patients within 6 years HD vintage showed that the two groups had similar volume status. In view of quite a few patients within 6 years HD vintage on twice-weekly HD still had some urine output in this study, we speculated that the above phenomenon is likely due to HD patients gradually decline of RKF with vintage advance. Thus, our study indicated that fluid management is a tough task for HD patients especially for those with less HD frequency, a timely switch from twice-weekly HD to thrice-weekly HD and an incremental dialysis strategy is important for HD patients.

Of note, despite that twice-weekly HD tended to have worse hydration status than that of thrice-weekly HD, we found that the two groups had similar survival, this result was consistent with many previous studies [6,7,28]. Several factors might contribute to the result: Firstly, a relatively higher rate of patients with maintained urine output in twice-weekly HD group would favor this treatment; secondly, a higher SpKt/V,a longer HD session time and a shorter HD vintage also benefited twice-weekly HD in the study. Moreover, the comparison between the two groups was not a “fair” competition. Few patients in thrice-weekly HD group initiation their hemodialysis therapy with twice-weekly HD and transferred to thrice-weekly HD later, whereas none of twice-weekly HD patients transferred from thrice-weekly HD.

The limitations of the present study include its design with a retrospective, but not a prospective cohort study. Besides, this is a single-center study with relative small number of patients, the sample size limitation might weaken the statistical power. Thirdly, we did not have precise RKF data, most patients in the study were in anuric stage, further studies for incident patients with certain RKF would be required. In spite of these shortcomings, we first reported the volume status of patients with twice-weekly hemodialysis, and we found a tendency of overhydration in this treatment model. Our finding would provide a new insight for volume management and would be meaningful for incremental dialysis strategy in HD patients.

## Conclusions

Twice-weekly hemodialysis patients had worse volume status than that of thrice-weekly HD patients with time, fluid management is a tough task for HD patients especially for those with less HD frequency and long-term dialysis vintage. BNP level was a powerful predictor of mortality in HD patients regardless twice-weekly HD or thrice-weekly HD.
